# 

**Published:** 1917-09-08

**Authors:** 


### CONTENTS.

| | PAGE |
|---|---|
| **Medical Education in the Time of the Great War** | 451 |
| **The Teaching Value of Lectures** | 452 |
| The Spoken Word and the Printed Page. | |
| **Specialism and Graduate Work** | 454 |
| The True Line of Medical Progress. | |
| **Medical Education in Wartime** | 455 |
| The Difficulties of the Schools. | |
| **The Royal Naval Medical Service** | 457 |
| Its Claims and Advantages. | |
| Qualifications and Regulations. | |
| **The Royal Army Medical Corps** | 458 |
| How Matters Stand To-day. | |
| **Dentistry as a Profession** | 461 |
| Its Drawbacks and Preparations to Qualify. | |
| **The Book Market** | 462 |
| **Woman's Work in Medicine After the War** | 463 |
| Memory Pictures and the Worker. | |
| **The Public Health Services** | 464 |
| Their Attraction for Practitioners. | |
| **The London Hospital** | 465 |
| His Majesty's Visit. Many Difficulties Overcome. | |
| **The Irish Nursing Board** | 466 |
| **The Medical Curriculum** | 467 |
| Qualification and Registration. | |
| The English Conjoint Course. | |
| The Fellowship of the Royal College of Surgeons. | |
| London University Course. | |
| Oxford University Course. | |
| Cambridge University Course. | |
| Higher Diplomas. | |
| **The Medical Student and His Work** | 470 |
| The Medical Schools of the United Kingdom. | |
| **The Public Services** | 476 |
| **Graduate Institutions in London** | 477 |
| **Tropical Medicine** | 477 |
| **Graduate Study in London Special Hospitals** | 478 |

